# Overcoming cut-off restrictions in multimorbidity prevalence estimates

**DOI:** 10.1186/1471-2458-14-780

**Published:** 2014-08-01

**Authors:** Barbara M Holzer, Klarissa Siebenhuener, Matthias Bopp, Christoph E Minder

**Affiliations:** Division of Internal Medicine, University Hospital Zurich, Zurich, Switzerland; Institute of Social and Preventive Medicine, University of Zurich, Zurich, Switzerland; Horten Center, University Hospital Zurich, Zurich, Switzerland; Center of Competence Multimorbidity, University of Zurich, Zurich, Switzerland

**Keywords:** Comorbidity, Multimorbidity, Definition, Operationalization, Multiple chronic conditions, Systematic review

## Abstract

**Background:**

Presently, there is no consensus on how to define multimorbidity. In this paper we investigate the connection between prevalence estimates for two or more and three or more chronic conditions to improve comparability of multimorbidity studies with different cut-offs.

**Methods:**

In a systematic review of the literature published between January, 1990 and December, 2011, we found 52 suitable studies, many providing prevalence estimates for several age groups. A total of 31 studies reported both the prevalence for multimorbidity based on two or more chronic conditions and three or more chronic conditions, which were analysed in this study. Our research question was whether there is a systematic interrelation between these two prevalence estimates, and how this could be used to improve the comparability of studies on the burden of multimorbidity.

**Results:**

Actually, we found a tight relationship between the prevalence of two or more and three or more chronic conditions. Moreover, each of these estimates can be predicted from the other. I.e. the cut-offs of two or three for the number of chronic conditions produce essentially the same information on prevalence.

**Conclusions:**

Our study shows a way to enhance and improve the comparability of prevalence estimates from different multimorbidity studies.

**Electronic supplementary material:**

The online version of this article (doi:10.1186/1471-2458-14-780) contains supplementary material, which is available to authorized users.

## Background

Multimorbidity is commonly defined as the co-occurrence of more than one chronic disease or chronic medical conditions in one person. This concept was introduced by van den Akker in a theoretical article with the aim to distinguish multimorbidity from comorbidity, where the focus lies on an index disease [1].

Numerous studies in many countries on prevalence of multimorbidity have been based on this concept of co-occurrence of several chronic diseases [2–6]. However, due to the absence of a widely accepted operationalization of multimorbidity, the comparability of these studies remains questionable. Known factors influencing prevalence estimates include the patient’s age, the number and the exact list of chronic conditions considered, and the minimum number of conditions required [7]. As a consequence, estimates of the prevalence of multimorbidity vary widely, e.g. from 13.1% to 71.8% for two or more chronic conditions in the general population or from nearly 0% to 95% for three or more chronic conditions in primary care populations [8].

Nevertheless, the simple count of chronic conditions not only provides a measure of prevalence, but is also the most commonly used approach to express the burden of multimorbidity [9]. Other widely used measures such as the Charlson Index or the Cumulative Illness Rating Scale (CIRS) are more complex and need more detailed data [10, 11], making them less suitable for a systematic review.

So far, the majority of researchers did use a cut-off point of two chronic conditions, with notable exceptions [12–14]. About a quarter of the studies presented estimates of prevalence of multimorbidity based on cut-offs 2+ and 3+ conditions and about half of these studies presented additional cut-offs of 4+ or/and 5+ chronic conditions [4, 15, 16].

Our study had three principal aims. Based on the data available from the systematic review mentioned above, we wanted

1. to explore the relationship between the prevalence of two or more chronic conditions (P2+) and that of three or more chronic conditions (P3+)
2. to find factors affecting the prevalence of two or more chronic conditions (P2+), given the prevalence of three or more chronic conditions (P3+) in the same study
3. on the basis of the above, and since P2+ is most commonly reported, to find a way to estimate the prevalence of two or more chronic conditions (P2+) in studies where only the prevalence of three or more chronic conditions (P3+) is available.

## Methods

### Data collection

**Figure 1:**
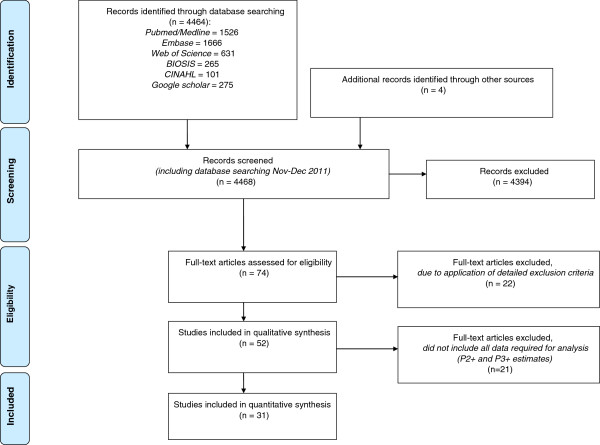
**PRISMA flow diagram of the evaluation process.**

Data for this study were obtained through a systematic review of the literature using MEDLINE/PreMEDLINE and Embase databases, CINAHL, the Web of Science and BIOSIS databases, and Google Scholar. We looked for relevant articles published in English or German between January, 1990 and December, 2011. For each database we used search strategies with the terms “multimorbidity” or “comorbidity” or “polymorbidity” and variations of these (see Additional file 1 for more information). Details regarding the search strategy and the criteria defined for evaluation were described elsewhere [17]. We included original studies addressing multimorbidity (no index-disease) with a sample size not smaller than 50. For better comparability, only study populations with a proportion of at least 50% Caucasians were included. The methods section had to satisfy scientific standards, in particular the chronic conditions selected and the prevalence of two or more (P2+) and three or more (P3+) chronic conditions estimates had to be identifiable. Studies were also included when one of these prevalence estimates was missing, but could be calculated from information given in the articles. Furthermore, the sample size of the study population as well as the description of the setting had to be reported. We compiled a database containing data of 52 studies. Figure 1 shows the flow diagram [18] according to PRISMA standards. We applied the PRISMA checklist as far as possible (see Additional file 2).

Most of the studies reported prevalence estimates for several age groups. A total of 31 studies reporting both, the prevalence of multimorbidity based on two or more (P2+) chronic conditions and three or more (P3+) chronic conditions remained for the present analysis, contributing 72 pairs of prevalence estimates, one for each age group or population in the study, including a total of about 10 million cases (details see Additional file 3). All 31 articles fulfilled at least 12 of the 22 possible quality items of the STROBE checklist (Strengthening the Reporting of Observational studies in Epidemiology) [19], as was previously suggested by Fortin [8].

In cases where explicit prevalence estimates per age group were missing and could not be derived from estimates given, we calculated prevalence based on information in the paper (e.g. size of the age group and number of multimorbid patients) or even in background papers referenced in the relevant study (15 articles). E.g. we added given estimates for 2, 3, 4, 5 and more chronic conditions together to get the two plus and three plus prevalence for each available age group.

For papers with only separate estimates by gender, we calculated weighted mean values and subsequently the overall mean per age group (4 articles). Furthermore, we obtained adequate estimates by calculating the size of the respective age group using the WHO-standard population (1 article) or by reading the prevalence of two plus and three plus chronic conditions out of graphs (1 article).

If mean age of the age group(s) was not indicated in the paper, it was derived from gender-specific one-year age-class general population data in the respective country and study period [20].

### Statistical analysis

Prevalence data for two or more (P2+) as well as three and more (P3+) chronic conditions were extracted from all studies in the systematic review reporting both prevalence estimates. These 72 pairs of prevalence estimates were converted into percentages and compared graphically. To obtain an adequate model for predicting logit P2+, we used a modified version of backward elimination to select the variables to be retained. Beginning with a full model including 8 factors and 30 parameters, we successively eliminated non-significant factors and grouped levels with similar effects within factors, simultaneously checking the criteria t^2^, I^2^, R^2^ (adjusted) and model F. We ended up with 2 competing models, one with 5 and one with 6 parameters. For the purpose of prediction, we then chose the more parsimonious model with the 5 predictors. Data were prepared using Microsoft Excel®. Statistical analyses and graphs were done with Stata 12.1. Model fitting was done using the Stata metareg procedure.

## Results

A total of 31 studies reported estimates of prevalence of P2+ and P3+ chronic conditions for a total of 72 prevalence groups (populations or age groups). Thus, the 31 studies contributed 72 pairs of prevalence estimates, each pair consisting of an estimate for P2+ and P3+ chronic conditions. A table presenting salient information regarding the studies and age groups included in the analysis is given in Additional file 3.

**Figure 2:**
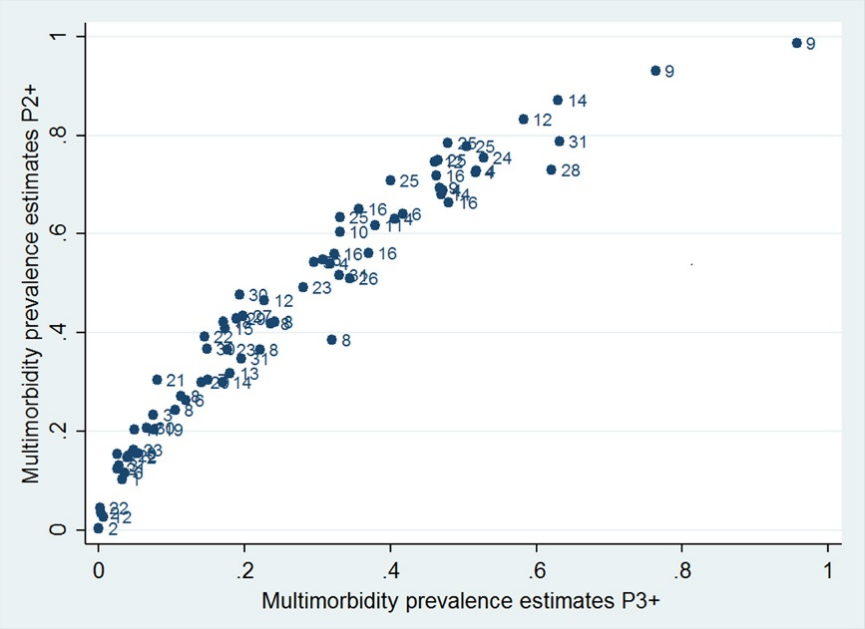
**Prevalence of multimorbidity in 72 prevalence groups (populations or age groups) from 31 studies.** All studies with prevalence estimates for two or more (P2+) and three or more (P3+) chronic conditions were shown. The index numbers give the number of the study from which the prevalence estimates originated (see Additional file 3).

Figure 2 presents the first main finding: There is a tight relationship between the prevalence estimates of two or more versus three or more chronic conditions, despite obvious and very substantial variation between studies in populations, definitions, study designs etc.

The tight relationship visible in Figure 2 permitted us to fit a statistical model relating P2+ to P3+. An initial model for predicting P2+ was set up with the following variables as predictors: P3+, study setting (GP practice, general population, health insurance), duration of study (0 to 10 yrs), number of age groups in study (1 to 5), span of age group (4 to 90 yrs, average 29.0), mean age of age group (12.8 yrs to 89.3 yrs, mean 59.5), data quality (rated as original data, or as calculated from data presented in the paper or as estimates read out from graphs), number of chronic conditions considered (5 up to more than 300), type of classification (ICD, ICPC-2, other).

Using these variables, a model suitable for predicting logit P2+ was found by elimination of insignificant variables and coarser grouping if there were only small effect differences between adjacent levels of the finer grouping of class variables. The continuous variables mean age of age group and P3+ were also squared. The final model contained the predictors logit P3+, mean age, whether the number of age groups exceeded 2 or not, and whether the number of conditions in the list considered was less than 20, between 20 and 99, or 100 or larger (Table 1).

**Table 1:** **Random effects logit model relating the prevalence of P2+ or P3+**

Predictor for logit P2+	Coefficient	Std.Error	t-value	p-value
Constant	0.04704	0.17429	0.27	0.788
Logit P3+	0.74285	0.02802	26.52	<0.0005
Nr of age groups > 2	0.23345	0.07435	3.14	0.003
Mean age	0.01055	0.002387	4.42	<0.0005
20-99 chronic conditions	-0.14314	0.084165	-1.7	0.094
≥100 chronic conditions	0.30834	0.11928	2.59	0.012

N = 72 prevalence groups (populations or age groups) from 31 studies.

Legend: logit P2 + = logit prevalence of 2 or more chronic conditions.

logit P3 + = logit prevalence of 3 or more chronic conditions.

The model fit was good: F (5, 66) = 322, R^2^ = 97.7% (adjusted), excess variability due to heterogeneity τ = 0.4068. The standard error of prediction (logit scale) of this model was 0.219 and essentially constant over the range, leading to the prediction limits and best predictions shown in Table 2.

**Table 2:** **Best predictions and 95% prediction intervals derived from model**

Predicted (logit)	logit P2+		P2+		
(best fit)	Low	High	Low	Best	High
-5	-5.43	-4.57	0	0.007	0.01
-4	-4.43	-3.57	0.01	0.018	0.027
-3	-3.43	-2.57	0.03	0.047	0.071
-2	-2.43	-1.57	0.08	0.119	0.172
-1.5	-1.93	-1.07	0.13	0.182	0.255
-1	-1.43	-0.57	0.19	0.27	0.36
-0.5	-0.93	-0.07	0.28	0.38	0.48
0	-0.43	0.43	0.39	0.5	0.61
0.5	0.07	0.93	0.52	0.62	0.72
1	0.57	1.43	0.64	0.73	0.81
1.5	1.07	1.93	0.74	0.82	0.87
2	1.57	2.43	0.83	0.881	0.919
3	2.57	3.43	0.93	0.953	0.969
4	3.57	4.43	0.97	0.982	0.988
5	4.57	5.43	0.99	0.993	0.996

I. e. in the worst situation (predicted logit = 0) the width of the prediction interval for the prevalence is 22% (39% to 61%). For all other values, the width is narrower. Using Table  2, studies providing P3+ only and not P2+ can now be compared to studies giving P2+ only. For small and large values of P3+ this comparison is precise, while for middle values of P3+ around 0.5 the precision is lower.

To estimate a value for P2+ when only P3+ is given, proceed as follows. First, compute the value of the logit predictor applicable to the study using Table 1. With the value so computed, enter Table 2 in the appropriate line. Interpolation is acceptable. Example: A study in question with three age groups and based on 35 chronic conditions provided P3 + = 0.2 for the age group 20-39 (mean age of 30 years). Logit P3 + = logit 0.2 is -1.3863 and from Table 1, the linear predictor of logit P2+ is 0.04704 + (0.74285*(-1.3863)) + 0.23345 + (0.01055*30) + (-0.14314) = -0.57596.

Using Table 2 with the value -0.576, one obtains a best estimate of P2+ of 0.36, and a 95% interval of prediction from 0.26 to 0.46 (lower to upper limit).

## Discussion

The simple count of chronic conditions is still the most common approach to express and measure the burden of multimorbidity [9]. In our study a basic element of this approach was examined: The cut-off point of two vs. three chronic conditions. To our knowledge, so far no study investigated this topic. One other article (a systematic review) did consider this point but not with statistical methods [8]. Our analysis shows that the relationship between P2+ and P3+ is influenced by the factors age, number of age groups and size of the list of chronic conditions considered in the study; in our data set, P3+ was the most influential factor, followed by mean age, the number of age groups in the study and the number of chronic conditions in the list. Number of age groups and number of chronic conditions were of about equal importance. Likely, the number of age groups in the study is a proxy for several effects: a small number of age groups may mean that only one or two wide age groups were considered. Alternatively, it could mean that the study gives only an age selected view of the situation.

Other proposed indices to measure the burden of multimorbidity are the Charlson Index [21], the Cumulative Illness Rating Scale [22], or concepts used in specific software to evaluate quality of outcomes, resource utilization such as the Johns Hopkins University Adjusted Clinical Groups (ACG®) Case-Mix System [23]. These measures are substantially more complex and geared to different purposes, thus not useful to determine the prevalence of multimorbidity in a population.

Our study has several weaknesses. As in any systematic review, not all factors of interest can be investigated, the notable example here being gender. Only very few studies were either covering one gender only, or looking at both gender separately.

The studies considered varied considerably in the quality of their reporting and probably execution. E.g. the population investigated was often described very sketchily, sometimes even the number of patients per age group was missing. The tight relationship presented in Figure 2 is quite surprising in view of the considerable heterogeneity of studies. We decided to use the simplest possible prediction model. As Figure 2 shows, nevertheless this model is fitting rather well. We therefore expect that the predictions derived from it to prove reliable. Our approach is not the only one feasible. Of course, one could also predict P3+ from P2+. Another approach would be to develop a joint prediction model for P2+ and P3+ and to use Bayesian methods to predict P2+ when only P3+ was known (and vice versa).

So far, most authors have used a cut-off of 2+ for the number of chronic conditions. In order to exploit studies with only a cut-off of 3+, we added Table 2 which helps finding an approximate corresponding prevalence for a cut-off of 2+, given a cut-off of 3+. In this paper we present evidence that cut-offs 2+ and 3+ for the number of chronic conditions provide essentially the same information on prevalence. If the prevalence estimates for a cut-off of 3+ chronic conditions is known, the prevalence for 2+, and possibly other cut-offs, may be estimated and vice versa. Hence, if one is interested in comparing prevalence estimates of multimorbidity, much of the discussion on whether a cut-off of 2+ or 3+ chronic conditions is more appropriate [8] becomes redundant.

In the broader discussion on how to operationalize and measure multimorbidity, many factors were mentioned, such as age, gender, the number and types of chronic conditions included, and factors concerning the setting and data collection methods [7–9, 24]. Our study established that, apart from the cut-off, the number of chronic conditions included as well as mean age and the number (and possibly the width) of age groups in the study, are of importance. We see great potential for improving comparability by agreeing on the most important factors to be routinely reported.

## Conclusions

Our study is a step towards addressing the problem of the comparability of prevalence estimates in the context of determining the burden of multimorbidity in a population. In a next step, other fundamental determinants of the prevalence (setting, age, gender, geographical area) have to be examined in order to contribute to the discussion on how to define and operationalize multimorbidity in epidemiological research.

## Authors’ informations

BMH senior research associate, epidemiology and public health.

KS postgraduate fellow, epidemiology and medicine.

MB senior research associate, head of the unit demographics and health statistics.

CEM emeritus professor of biostatistics of the University of Bern.

## Electronic supplementary material

Additional file 1: **Database search terms.** (PDF 54 KB)

Additional file 2: **PRISMA checklist.** (PDF 84 KB)

Additional file 3: **Studies included in the analysis (N = 31) by age groups with prevalence estimates and complete reference information for studies listed in the table.** (PDF 44 KB)
