# Supplementary material for: Overcoming cut-off restrictions in multimorbidity prevalence estimates
Source: BMC Public Health. 2014 Aug 1;14:780. doi: 10.1186/1471-2458-14-780 (PMC4133617; doi:10.1186/1471-2458-14-780)
Supplement: Supplementary file 1 — Additional file 1: Database search terms. (PDF 54 KB) [file 12889_2014_6931_MOESM1_ESM.pdf]

Additional file 1 – Search terms

As run in Pubmed/Medline and other electronic databases

1. multimorbidity
2. multimorbidities
3. multi-morbidity
4. multi-morbidities
5. multimorbiditie
6. multi-morbiditie
7. „multimorbidity's"
8. „multi-morbidity's"
9. multimorbid
10. multi-morbid
11. comorbidity
12. co-morbidity
13. comorbiditie
14. comorbidities
15. co-morbidities
16. comorbid
17. co-morbid
18. "comorbidity's"
19. "co-morbidity's"
20. "multiple chronic condition"
21. "multiple chronic conditions"
22. "multiple chronic illness"
23. "multiple chronic disease"
24. "multiple chronic diseases"
25. "chronic medical condition "
26. "chronic medical conditions "
27. "chronic clinical condition"
28. "chronic clinical conditions"
29. polymorbidity
30. "polymorbidity's"
31. poly-morbidity
32. "poly-morbidity's"
33. polymorbiditie
34. poly-morbiditie
35. polymorbidities
36. poly-morbidities
37. polymorbid
38. poly-morbid

Limited to English or German language and time period 1990 – 2011.
