# Supplementary material for: Overcoming cut-off restrictions in multimorbidity prevalence estimates
Source: BMC Public Health. 2014 Aug 1;14:780. doi: 10.1186/1471-2458-14-780 (PMC4133617; doi:10.1186/1471-2458-14-780)
Supplement: Supplementary file 3 — Additional file 3: Studies included in the analysis (N = 31) by age groups with prevalence estimates and complete reference information for studies listed in the table. (PDF 44 KB) [file 12889_2014_6931_MOESM3_ESM.pdf]

Additional file 3: Studies included in the analysis (N = 31) by age groups with prevalence estimates.

Study nr.	Name (Year)	Country Study Population	Data source / study	Nr. of patients	Nr. of age groups	Age group	Prevalence P2+	Prevalence P3+
1	Newacheck et al 1991	United States	National health interview survey on child health	7'465	1	10-17	10.2	3.2
2	Schellevis et al 1993	The Netherlands	database of 7 GP practices	23'534	2	<65	0.3	0
						≥65	3.5	0.3
3	Menotti et al 2001	Finland	Different cohorts of FINE Study	716	1	65-84	23.3	7.5
		The Netherlands		887	1	65-84	13.1	2.8
		Italy		682	1	65-84	15.3	2.5
4	Wolff et al 2002	United States	Medicare, Part A and B	1'217'103	5	65-69	53.9	31.7
						70-74	63.1	40.6
						75-79	68.8	47.2
						80-84	72.5	51.6
						≥85	72.8	51.8
5	Chan et al 2002	Australia	Survey of hospital admissions, Randwick Area	526	1	55-95	54.2	29.5
6	Rapoport et al 2004	Canada	National population health survey	13'682	4	20-39	11.5	3.5
						40-59	26.3	11.9
						60-79	54.7	30.7
						≥80	64.0	41.7
7	Macleod et al 2004	United Kingdom	Cairns practice registry, Glasgow	7'286	1	≥18	30.4	15
8	Alonso et al 2004	United States	General social survey	2'474	1	mean 43.6	41.8	23.6
		Denmark	Danish health survey	4'084	1	≥16	15.5	5.3
		France	Household survey	3'656	1	≥15	27.1	11.3
		Germany	Infratest national database	2'914	1	≥14	36.5	22.1
		Italy	National sample from electoral lists	2'031	1	≥18	42.2	24.1
		The Netherlands	National and Amsterdam registry	4'059	1	≥12	38.6	32
		Norway	National registry	2'323	1	19-80	24.3	10.5
9	Fortin et al 2005	Canada	21 GPs of Saguenay, Quebec	980	3	18-44	69.3	46.7
						45-64	93.0	76.4
						≥65	98.7	95.7
10	Naughton et al 2006	Ireland	National pharmacy claims database	316'928	1	≥70	60.4	33.1
11	Min et al 2007	United States	ACOVE study	372		≥65	61.8	37.9
12	Britt et al 2008	Australia	General practice substudy of BEACH program	9'111	5	<25	2.6	0.6
						25-44	14.7	3.9
						45-64	46.5	22.7
						65-74	74.6	46.0
						≥75	83.2	58.2
13	Hudon 2008	Canada	Quebec health survey	16'782	1	18-69	31.8	18
14	Uijen et al 2008	The Netherlands	CMR, GP registry in Nijmegen	13'584	3	45-64	29	17
						65-74	66	47
						>75	85	72
15	Lee et al 2008	United States	Veteran health care database	741'847	1	55-64	40.8	17.3
16	Schram et al 2008	The Netherlands	Leiden 85-plus population-based Study	599	1	≥85	65	35.6
			Rotterdam population-based Study	3'550	1	65-99	71.8	46.3
			LASA population-based Study	2'463	1	≥55	56	32.3
			CMR, GP registry in Nijmegen	2'895	1	≥55	66.3	48
			RNUGP, GP registry Leiden region	5'610	1	≥55	56.1	37
17	Wang et al 2008	Germany	Health survey (20 GPs)	1'009	1	15-89	20.3	4.9
18	Murtaugh et al 2009	United States	OASIS data set (MediCare, MediCaid)	5'585'931	1	≥65	42.1	17.1
19	Schneider et al 2009	United States	CCW data (MediCare)	1'649'574	1	≥0	20.3	7.6
20	Loza et al 2009	Spain	EPISER Health survey	2'192	1	≥20	30	14
21	Minas et al 2010	Central Greece	Data from various primary health care centers	20'299	2	<65	12.4	2.5
						≥65	30.4	8.0
22	Taylor et al 2010	South Australia	North West Adelaide health study	3'203	3	20-39	4.4	0.2
						40-59	15.0	4.1
						60-90	39.2	14.5
23	Gunn et al 2010	Australia	Diamond longitudinal study (various GPs)	6'864	3	18-44	16.2	4.8
						45-64	36.5	17.6
						≥65	49.1	28.0
24	Jansa et al 2010	Spain	Survey of hospital discharges, Barcelona	301	1	23-93	75.4	52.8
25	Hung et al 2011	United States	Health and retirement study 2008	11'321	5	65-69	63.4	33.1
						70-74	70.8	40.0
						75-79	75.0	46.5
						80-84	78.5	47.9
						≥85	77.8	50.4
26	Aarts et al 2011	The Netherlands	Postal survey, Limburg	15'188	1	55-90	50.9	34.4
27	Wong et al 2011	The Netherlands	Dutch hospital register	1'414'142	1	≥0	43.4	19.8
28	v.d. Bussche et al 2011	Germany	GEK claims database	123'224	1	≥65	73	62.1
29	Galenkamp et al 2011	The Netherlands	LASA population-based study	2'046	1	57-98	42.8	18.9
30	Tucker-Seeley et al 2011	United States	Health and retirement study 2004	7'305	3	50-59	20.7	6.5
						60-69	36.8	14.8
						≥70	47.6	19.3
31	Naessens et al 2011	United States	Various administrative data	33'324	3	18-34	34.8	19.5
						35-49	51.6	33.0
						50-64	78.8	63.2

Additional file 3 - References (as supplied by the authors)

1. Newacheck PW, McManus MA, HB. F: **Prevalence and impact of chronic illness among adolescents.** *Am J Dis Child* 1991, **145**(12):1367-1373.
2. Schellevis FG, van der Velden J, van de Lisdonk E, van Eijk JT, C. vW: **Comorbidity of chronic diseases in general practice.** *J Clin Epidemiol* 1993, **46**(5):469-473.
3. Menotti A, Mulder I, Nissinen A, Giampaoli S, Feskens EJM, Kromhout D: **Prevalence of morbidity and multimorbidity in elderly male populations and their impact on 10-year all-cause mortality: The FINE study (Finland, Italy, Netherlands, elderly)**. *Journal of Clinical Epidemiology* 2001, **54**(7):680-686.
4. Wolff JL, Starfield B, Anderson G: **Prevalence, expenditures, and complications of multiple chronic conditions in the elderly**. *Arch Intern Med* 2002, **162**(20):2269-2276.
5. Chan DK, Chong R, Basilikas J, Mathie M, Hung WT: **Survey of major chronic illnesses and hospital admissions via the emergency department in a randomized older population in Randwick, Australia**. *Emerg Med (Fremantle)* 2002, **14**(4):387-392.
6. Rapoport J, Jacobs P, Bell NR, Klarenbach S: **Refining the measurement of the economic burden of chronic diseases in Canada**. *Chronic Dis Can* 2004, **25**(1):13-21.
7. Macleod U, Mitchell E, Black M, G. S: **Comorbidity and socioeconomic deprivation: an observational study of the prevalence of comorbidity in general practice.** *Eur J Gen Pract* 2004, **10**(1):24-26.
8. Alonso J, Ferrer M, Gandek B, Ware JE, Jr., Aaronson NK, Mosconi P, Rasmussen NK, Bullinger M, Fukuhara S, Kaasa S *et al*: **Health-related quality of life associated with chronic conditions in eight countries: results from the International Quality of Life Assessment (IQOLA) Project**. *Qual Life Res* 2004, **13**(2):283-298.
9. Fortin M, Bravo G, Hudon C, Vanasse A, Lapointe L: **Prevalence of multimorbidity among adults seen in family practice**. *The Annals of Family Medicine* 2005, **3**(3):223-228.
10. Naughton C, Bennett K, J. F: **Prevalence of chronic disease in the elderly based on a national pharmacy claims database.** *Age Ageing* 2006, **35**(6):633-636.
11. Min LC, Wenger NS, Fung C, Chang JT, Ganz DA, Higashi T, Kamberg CJ, MacLean CH, Roth CP, Solomon DH *et al*: **Multimorbidity is associated with better quality of care among vulnerable elders**. *Medical Care* 2007, **45**(6):480-488.
12. Britt HC, Harrison CM, Miller GC, Knox SA: **Prevalence and patterns of multimorbidity in Australia**. *Med J Aust* 2008, **189**(2):72-77.
13. Hudon C, Soubhi H, Fortin M: **Relationship between multimorbidity and physical activity: secondary analysis from the Quebec health survey**. *BMC Public Health* 2008, **8**:304.
14. Uijen AA, van de Lisdonk EH: **Multimorbidity in primary care: prevalence and trend over the last 20 years**. *Eur J Gen Pract* 2008, **14 Suppl 1**:28-32.
15. Lee TA, Shields AE, Vogeli C, Gibson TB, Woong-Sohn M, Marder WD, Blumenthal D, Weiss KB: **Mortality rate in veterans with multiple chronic conditions**. *J Gen Intern Med* 2007, **22 Suppl 3**:403-407.
16. Schram MT, Frijters D, van de Lisdonk EH, Ploemacher J, de Craen AJ, de Waal MW, van Rooij FJ, Heeringa J, Hofman A, Deeg DJ *et al*: **Setting and registry characteristics affect the prevalence and nature of multimorbidity in the elderly**. *J Clin Epidemiol* 2008, **61**(11):1104-1112.

17. Wang H-M, Beyer M, Gensichen J, Gerlach FM: **Health-related quality of life among general practice patients with differing chronic diseases in Germany: Cross sectional survey**. *BMC Public Health* 2008, **8**(1):246.
18. Murtaugh C, Peng T, Totten A, Costello B, Moore S, H. A: **Complexity in geriatric home healthcare.** *J Healthc Qual* 2009, **31**(2):34-43.
19. Schneider KM, O'Donnell BE, Dean D: **Prevalence of multiple chronic conditions in the united states' medicare population**. *Health Qual Life Outcomes* 2009, **7**:82.
20. Loza E, Jover JA, Rodriguez L, Carmona L: **Multimorbidity: prevalence, effect on quality of life and daily functioning, and variation of this effect when one condition is a rheumatic disease**. *Seminars in arthritis and rheumatism* 2009, **38**(4):312-319.
21. Minas M, Koukosias N, Zintzaras E, Kostikas K, Gourgoulianis KI: **Prevalence of chronic diseases and morbidity in primary health care in central Greece: An epidemiological study**. *BMC Health Services Research* 2010, **10**(1):252.
22. Taylor AW, Price K, Gill TK, Adams R, Pilkington R, Carrangis N, Shi Z, Wilson D: **Multimorbidity - not just an older person's issue. Results from an Australian biomedical study**. *BMC Public Health* 2011, **10**(1):718.
23. Gunn JM, Ayton DR, Densley K, Pallant JF, Chondros P, Herrman HE, Dowrick CF: **The association between chronic illness, multimorbidity and depressive symptoms in an Australian primary care cohort**. *Soc Psychiatry Psychiatr Epidemiol* 2012.
24. Jansà M, Hernández C, Vidal M, Nuñez M, Bertran MJ, Sanz S, Castell C, Sanz G: **Multidimensional analysis of treatment adherence in patients with multiple chronic conditions. A cross-sectional study in a tertiary hospital**. *Patient Education and Counseling* 2010, **81**(2):161-168.
25. Hung WW, Ross JS, Boockvar KS, Siu AL: **Recent Trends in Chronic Disease, Impairment and Disability Among Older Adults in the United States**. *BMC Geriatrics* 2011, **11**(1):47.
26. Aarts S, van den Akker M, Hajema KJ, van Ingen AM, Metsemakers JFM, Verhey FRJ, van Boxtel MPJ: **Multimorbidity and its relation to subjective memory complaints in a large general population of older adults**. *International Psychogeriatrics* 2011, **23**(04):616-624.
27. Wong A, Boshuizen HC, Schellevis FG, Kommer GJ, Polder JJ: **Longitudinal administrative data can be used to examine multimorbidity, provided false discoveries are controlled for**. *Journal of Clinical Epidemiology* 2011, **64**(10):1109-1117.
28. Van den Bussche H, Koller D, Kolonko T, Hansen H, Wegscheider K, Glaeske G, von Leitner EC, Schafer I, Schon G: **Which chronic diseases and disease combinations are specific to multimorbidity in the elderly? Results of a claims data based cross-sectional study in germany**. *BMC Public Health* 2011, **11**(1):101.
29. Galenkamp H, Braam AW, Huisman M, Deeg DJH: **Somatic Multimorbidity and Self-rated Health in the Older Population**. *The Journals of Gerontology Series B: Psychological Sciences and Social Sciences* 2011, **66B**(3):380-386.
30. Tucker-Seeley RD, Li Y, Sorensen G, Subramanian SV: **Lifecourse socioeconomic circumstances and multimorbidity among older adults**. *BMC Public Health* 2011, **11**(1):313.
31. Naessens JM, Stroebel RJ, Finnie DM, Shah ND, Wagie AE, Litchy WJ, Killinger PJ, O'Byrne TJ, Wood DL, RE. N: **Effect of multiple chronic conditions among working-age adults.** *Am J Manag Care* 2011, **17**(2):118-122.
